# Disruption of Metapopulation Structure Reduces Tasmanian Devil Facial Tumour Disease Spread at the Expense of Abundance and Genetic Diversity

**DOI:** 10.3390/pathogens10121592

**Published:** 2021-12-08

**Authors:** Rowan Durrant, Rodrigo Hamede, Konstans Wells, Miguel Lurgi

**Affiliations:** 1 Department of Biosciences, Swansea University, Singleton Park, Swansea SA2 8PP, UK; r.durrant.1@research.gla.ac.uk (R.D.); k.l.wells@swansea.ac.uk (K.W.); 2 School of Natural Sciences, University of Tasmania, Hobart, TAS 7001, Australia; rodrigo.hamedeross@utas.edu.au

**Keywords:** dispersal, contact distance, landscape-scale genetic diversity, disease transmission, disease management, metapopulation networks, metapopulation disease dynamics, fragmentation

## Abstract

Metapopulation structure plays a fundamental role in the persistence of wildlife populations. It can also drive the spread of infectious diseases and transmissible cancers such as the Tasmanian devil facial tumour disease (DFTD). While disrupting this structure can reduce disease spread, it can also impair host resilience by disrupting gene flow and colonisation dynamics. Using an individual-based metapopulation model we investigated the synergistic effects of host dispersal, disease transmission rate and inter-individual contact distance for transmission, on the spread and persistence of DFTD from local to regional scales. Disease spread, and the ensuing population declines, are synergistically determined by individuals’ dispersal, disease transmission rate and within-population mixing. Transmission rates can be magnified by high dispersal and inter-individual transmission distance. The isolation of local populations effectively reduced metapopulation-level disease prevalence but caused severe declines in metapopulation size and genetic diversity. The relative position of managed (i.e., isolated) local populations had a significant effect on disease prevalence, highlighting the importance of considering metapopulation structure when implementing metapopulation-scale disease control measures. Our findings suggest that population isolation is not an ideal management method for preventing disease spread in species inhabiting already fragmented landscapes, where genetic diversity and extinction risk are already a concern.

## 1. Introduction

Infectious diseases are a major threat to the long-term survival of wildlife populations across many taxa [1]. Infectious cancers in wildlife are nowadays recognised as a major conservation problem, particularly for endangered species and populations with restricted distribution [2,3]. Genetic bottlenecks, changes in global climate and anthropogenic activities resulting in exposure to toxins, oncogenic pathogens, immunosuppression, stress and urbanisation have been regarded as contributors to the emergence of cancers in wildlife [4,5,6]. As well as threatening biodiversity, wildlife diseases are increasingly being acknowledged as potential risks to human health and domestic livestock [7,8], leading to substantial economic loss and compromising human wellbeing [9,10].

The management of wildlife diseases is often carried out at the individual or local population level [11]. However, transmission between local populations is a fundamental mechanism of infectious disease spread at intermediate to large spatial scales [12,13,14], and has been identified as one of the main causes of epidemics in both humans [15] and animals [16], including the recent global spread of COVID-19 [17,18,19]. Increased host movement has been shown to drive both the regional spread of disease [13,14,20] and host extinction risk from disease in theoretical metapopulations (i.e., collections of local populations connected via dispersal corridors) [14,21]. The metapopulation structure of the host species can also shape the source-sink dynamics of both the host and the disease, allowing host populations with low reproduction rates to benefit from migrants from populations above their carrying capacity, and the disease to persist in populations where the R_0_ is below 1 [22,23]. However, predicting the influence of metapopulation structure on the dynamics of disease-burdened host populations is not a trivial task, particularly for emerging infectious diseases with high mortality. Restricting the movement of individual animals in order to reduce the spread of disease is tempting, but it could result in great ecological costs, such as disrupting migratory patterns and reducing gene flow between populations, as barriers that reduce disease spread have also been shown to shape landscape genetics [24]. The success of containing infectious disease spread at landscape scales may depend crucially on the connectivity between local populations. While increasing connectivity may facilitate efficient disease spread and disease-induced host population decline, reduced population connectivity may disrupt vital patch colonization dynamics and the maintenance of host genetic diversity [21,25].

Devil facial tumour disease (DFTD) is a transmissible cancer that has decimated populations of Tasmanian devils (*Sarcophilus harrisii*) across nearly the entire geographical range of the species [26,27]. The transmission of DFTD between individuals occurs when infected and susceptible individuals bite each other, a common behaviour in this species [28]. DFTD is able to evade host immune detection through epigenetic downregulation of major histocompatibility complex (MHC) gene expression in tumour cells [29]. DFTD prevalence is considerably high (up to 80%) even after populations have suffered significant declines, suggesting that transmission is mostly frequency dependent [30]. This is driven by the increase in contact rates and biting wounds during the mating season [31,32]. The spread of DFTD across Tasmania is particularly worrying due to the large declines in numbers associated with it, with an average decline in affected populations of 77% [33], and some local populations having declined by as much as 90% [34]. Landscape-scale spread of the disease occurred relatively fast, with the disease now found on over 90% of the landmass of Tasmania [27].

Previous work has generated a good understanding of how DFTD spreads between individuals, how it leads to population decline and how it drives demographic changes within local populations [30,35,36,37,38]. However, we still know surprisingly little about the processes that drive the spread of DFTD on a regional scale. Counter to the findings of previous single-population models of DFTD that predicted a high probability of host extinction [30], a recent metapopulation modelling study [39] found that the recolonization of extinct local devil populations in a metapopulation may be strong enough to prevent their complete extinction. This suggests that metapopulation dynamics play an important role in maintaining devil populations. Given their recognised instrumental role in maintaining landscape-scale population viability and in driving disease spread, we expect metapopulation structure and dynamics to play a fundamental role in DFTD transmission across devil populations in Tasmania.

Here we aim at unveiling the role of local-scale processes, such as within-population mixing and transmission, and their interaction with regional-scale processes of between-population dispersal, in driving the regional spread of DFTD in structured metapopulations. We developed an individual-based metapopulation model of DFTD spread and investigated the effects of altering the magnitude of within-population mixing, devil-to-devil disease transmission and devils’ dispersal rate on metapopulation disease dynamics. We additionally explored the effects of disrupting metapopulation structure on disease dynamics by isolating local populations. This enabled us to assess whether metapopulation fragmentation via isolation methods such as fencing or geographic barriers would be a viable strategy for managing regional disease outbreaks and how it would affect the size and landscape-scale genetic diversity of devil populations.

We hypothesised that dispersal rate would have the largest influence over regional disease spread, as opposed to within-population mixing and disease transmission probability, by allowing for local outbreaks to become regional. We also hypothesised that local population isolation (i.e., metapopulation fragmentation) would lead to a decrease in disease prevalence and an increase in devil abundance at metapopulation scales relative to scenarios where no populations are isolated. This would, however, also cause a decrease in genetic diversity. Finally, we hypothesised that these effects would be most pronounced when isolating well-connected populations within the metapopulation network.

## 2. Methods

We developed an individual-based metapopulation model of DFTD spread in Tasmanian devils on the island of Tasmania, Australia. Metapopulation structure was defined as a network of local populations (nodes) connected via dispersal corridors (links) for devils to move/disperse across populations. Due to the challenge of incomplete landscape scale, devil surveillance across Tasmania, geographical location and size of local devil populations in our metapopulation was determined based on known suitable habitat for the species. Local population and disease transmission dynamics were modelled according to our previously developed model [38], parameterised by long-term detailed monitoring data [40,41].

Using this model, we performed computer simulations with different combinations of parameter values governing dispersal, inter-individual contact distance and disease transmission rate to investigate the factors driving regional disease spread. Further simulations were performed to assess the effects of isolating local populations (by removing links from selected nodes) as a management strategy on landscape-scale population size, disease prevalence and genetic diversity.

### 2.1. Metapopulation Structure

**Figure 1:**
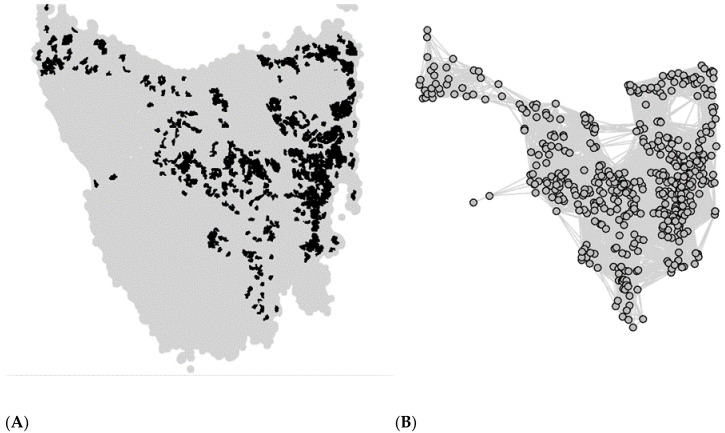
Tasmanian devil metapopulation network map and corresponding habitat. (**A**) Distribution of sclerophyll forest and coastal heath patches across Tasmania obtained from the TASVEG2 dataset [45]. Patches with an area size <5 km^2^ and those with estimated population density <0.5 devils/km^2^ were excluded. (**B**) The resulting metapopulation network constructed from A. Each node in the network represents a local population of devils, and each edge (i.e., a connection between two nodes) represents a dispersal corridor between the connected populations.

We defined metapopulation structure as a network of local populations connected according to their proximity to each other. Geographical location and size of local populations was determined using the position and area of patches of sclerophyll forests and coastal heath across Tasmania, where devil density was known to be reasonably high pre-DFTD to assume viable populations according to available devil surveillance data [42,43] and recently published devil density estimates [27] (see Supplementary Materials Methods S1 for full description). This yielded a total of 477 populations, with a mean habitat patch area of 13.3 km^2^ (minimum area 5.0 km^2^, maximum area 102.2 km^2^) (Figure 1; Supplementary Materials Figure S1b). Dispersal corridors between populations (i.e., the edges in the metapopulation network) were defined according to their relative geographical location. Any two populations within 50 km of each other were assumed to be connected, representing a balance between the largest dispersal events observed in devils (~110 km) and average estimates based on genetic and demographic analyses (~14–30 km) [44].

### 2.2. Individual-Based Model

Each local population in the metapopulation was comprised by a set of individual devils characterised by the following attributes: sex, age, infection status, tumour volume and location within the local population (x and y coordinates). During the course of model simulations, individuals were subjected to eight demographic and epidemiological processes in each modelled weekly time step (the time scale of the model) as originally presented in [38]: (1) reproduction and release of offspring into the local population, (2) movement within the current habitat patch (i.e., local population), (3) dispersal across local populations according to certain probability (*γ*), (4) aging, (5) non-DFTD related deaths governed by the carrying capacity of local populations, (6) infection of susceptible individuals governed by inter-individual contact distance (δ) and disease transmission rate (*β*), (7) tumour growth, and (8) DFTD-induced mortality determined by the size of the tumour (Figure S2). Full description of these demographic and epidemiological processes can be found in the Supplementary Materials methods section (Methods S1).

### 2.3. Tracing the Mixing of Individuals from Different Populations

To assess the extent to which metapopulation fragmentation (via population isolation) could potentially restrict gene flow between local devil populations, we traced between-population genetic mixing as a proxy for devil’s genetic diversity across the metapopulation. Individuals were assigned a population-specific “genotype” at the beginning of each simulation. Genetic inheritance during reproduction followed a simple model in which all adult males in a population have an equal probability of fathering local offspring. All offspring from a single brood were assumed to be fathered by the same male individual. For simplicity, offspring inherited either the mother or father’s genotype with equal probability. Population mixing was measured for each local population using Nei’s within-population variation index *H_i_* [46]

(1) $$H_{i}=1-\underset{g}{\sum }x_{ig}^{2}\;$$

where *x_ig_* is the proportion of individuals in population *i* with the genotype *g*. When a population was empty, its genetic variation was set to zero. This measure of population mixing was only used in habitat fragmentation experiments (see below).

### 2.4. Model Simulations

During each time step (1 week), the following events occurred in order: (1) dispersal of individuals between local populations in the metapopulation network, (2) movement of individuals within populations, (3) offspring release into the free-ranging population (once every 52 timesteps only), (4) death due to DFTD, (5) tumour growth, (6) disease transmission between individuals within specified contact distance, (7) non-DFTD deaths and (8) aging.

At the start of each simulation, local population size (i.e., number of individuals) was initialised independently for each population using population density estimates for the year 1986 [27], habitat patch area and the same density multiplier used to calculate carrying capacity (see Section 2.1; Supplementary Materials Table S1). Age, sex and location (i.e., coordinates in space) of each individual were randomly assigned. Age was uniformly sampled between 32 and 364 weeks; sex was randomly assigned with 0.5 probability; spatial coordinates (x and y, independently) were uniformly sampled between 0 to the square root of the population’s habitat patch area (i.e., the habitat patch was assumed to be square shaped). After a 520 week (i.e., ten years) burn-in period in the absence of disease to ensure the stability of metapopulation dynamics and demographics before the disease introduction, three randomly chosen individual devils from the population closest to the location where DFTD was first recorded in Tasmania [26] were infected with DFTD to minimise the risk of all initially infected individuals dying before the outbreak was established.

To investigate the effect of varying contact distance (δ), disease transmission coefficients (β) and the baseline dispersal rate (*γ*_0_) on population size, disease prevalence and population stability, we ran the model described above for a total of 1820 weekly time steps (35 years) for each combination of these parameters’ values (Supplementary Materials Table S1). Contact distance took values from 0.1 to 0.5 km, with increments of 0.1 km; disease transmission coefficients were varied between 0.2–0.8 increasing by 0.1; baseline dispersal rate values ranged from 0.001 to 0.01, with increments of 0.001 (a dispersal probability of 0.001 is equivalent to 520 dispersal events per 10,000 individuals per year). We performed 20 replicated simulations for each parameter combination, resulting in 7000 simulations in total.

### 2.5. Metapopulation Analysis

Metapopulation dynamics and disease spread resulting from the parameter combinations introduced above were assessed using two summary measures:

(1) Total metapopulation size: quantified as the median, across the last 520 weeks (ten years) of the simulation, of the sum of the sizes (i.e., number of individuals) of all local populations at each time step.
(2) Proportion of local populations where at least one case of DFTD was recorded during the entire simulated time period.

### 2.6. Pattern Matching of Disease Spread to Empirical Data

We identified the parameter combination from our simulations for which the spatial patterns of disease emergence most closely matched a recently published empirical prediction of the disease front from 1996 to 2015 [47] and predicted metapopulation size declines from 1986 to 2020 [27]. Parameter combinations were first filtered to match estimates of metapopulation size for the year 2020 from empirical data according to the 95% credible intervals of estimates reported by Cunningham et al. [27] and with a DFTD prevalence ≥5%. From the remaining parameter combinations, the one with the best match for disease arrival at different locations in Tasmania through time (assessed within five-year time windows of empirical estimates [47]) was assumed to represent the most realistic simulation scenario. Matching of disease arrival time between simulated and empirical data was quantified as the proportion of local populations falling within the correct temporal disease front as reported by [47]. The parameter combination thus selected was used for the metapopulation fragmentation experiments.

### 2.7. Metapopulation Fragmentation Experiments

To evaluate the consequences of population isolation/metapopulation fragmentation on population dynamics and disease spread, we ran a series of simulation experiments where up to 100 local populations were isolated from the metapopulation. Between 10 and 100 populations were isolated (incrementally in sets of 10; i.e., 10, 20, 30, …, 100) at week 1040 of each fragmentation simulation. We implemented three different isolation methods based on the connectivity profiles of local populations:

(1) Random: Populations to be isolated were randomly chosen.
(2) Degree: Isolated populations were chosen in order according to their degree (i.e., the number of connections of the local population), from the most connected to the least connected population.
(3) Betweenness: Populations to isolate were selected in order according to their betweenness centrality, a node-level network measure that quantifies the extent to which the shortest paths connecting any two nodes (populations) in the network (the metapopulation) comprise the focal node. Local populations were thus ranked from most to least central according to their betweenness centrality, and those with the highest centrality scores were removed first. Betweenness centrality of a local population *v* was calculated thus [48]

(2) $$B\left(v\right)=\underset{a\neq v\neq b}{\sum }\frac{\sigma_{avb}}{\sigma_{ab}}$$

where $\sigma_{ab}$ is the number of shortest paths that between population a and population b, and $\sigma_{avb}$ is the number of these that pass through population *v*.

We performed 30 replicated simulations for each isolation method and number of populations isolated, with DFTD being introduced 520 weeks (10 years) into the 1820-week (35 year) simulation. To compare the outcomes of the disease dynamics to the neutral (i.e., no disease) scenario, we performed a further 20 replicated simulations without DFTD being introduced. This resulted in 1500 simulations in total. Total metapopulation size, disease prevalence and proportion of local populations reached by DFTD were calculated as above. Additionally, the mean within-population genetic variation (Equation (1)) was quantified to assess the effects of fragmentation on genetic composition of the metapopulation.

Model implementation and analyses were carried out using R version 4.1 [49]. Source code for the full model and scripts used for data analyses is available from the GitHub repository: https://github.com/RowanDurrant/DFTD (accessed on 12 November 2021).

## 3. Results

### 3.1. Disease Spread Is Driven by the Interplay between Local Transmission and Regional Movement

**Figure 2:**
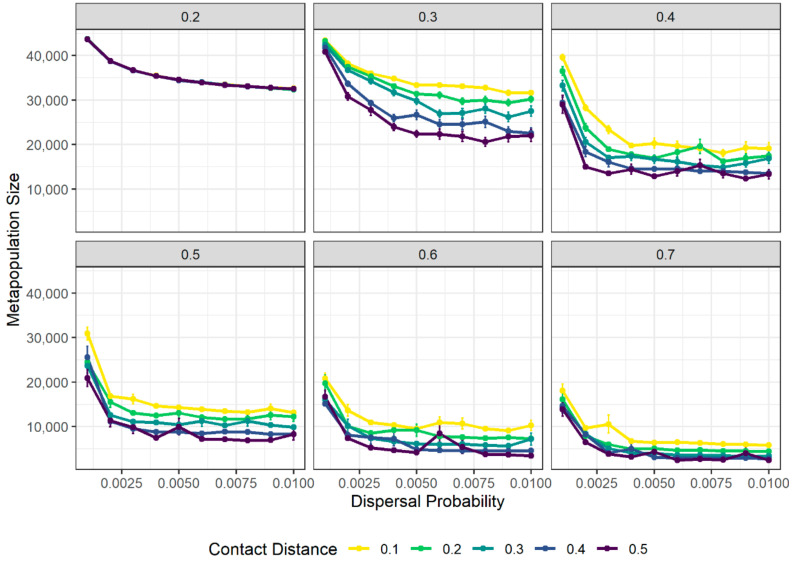
Tasmanian devil metapopulation size is influenced synergistically by within-population mixing (contact distance), dispersal rate and transmission probabilities. Points and vertical bars show the mean and standard error (across 20 replicated simulations) of median values of metapopulation size taken over 520 weeks (10 years) of each simulation. Panels display results for different transmission probability scenarios (numbers at the top of the panel). Colour of points and lines represent different contact distance scenarios.

Population size decreased as dispersal rate (*γ*_0_) increased across all values of transmission rate (β) and contact distance (δ). However, only under scenarios of relatively large transmission rate (β ≥ 0.5) did increasing contact distance and dispersal rate lead to reductions in overall metapopulation size to below Cunningham et al.’s [27] predictions of the 2020 devil metapopulation size (16,900 individuals) for the majority of parameter combinations (Figure 2). This suggests that all three factors interact synergistically to cause major population declines.

At relatively low transmission probability values (e.g., β = 0.2), metapopulation size declined as dispersal rate increased at the same rate across all values of contact distance. This pattern was also observed in the absence of disease (Supplementary Materials Figure S3), suggesting that this population decline is caused by source–sink metapopulation dynamics independent of disease effects.

**Figure 3:**
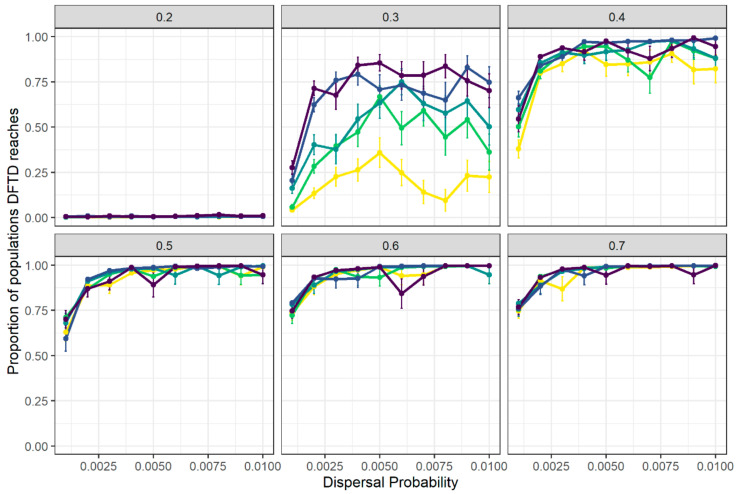
Average proportion of local devil populations reached by DFTD is influenced by contact distance, dispersal and transmission probabilities. Points and vertical bars show the means and standard error of median values of the fraction of local populations within the metapopulation reached by disease within the time period of the simulation, taken over the last 520 weeks (10 years) of each simulation. Panels display results for each value of transmission probability (numbers at the top of each panel). Point and line colours represent different values of contact distance.

At the transmission probability of 0.2, DFTD fails to spread far beyond the initially infected population, across all values of dispersal rate and contact distance (Figure 3). At higher values of disease transmission, however, the proportion of infected populations rises with the increase in dispersal probability. Contact distance only has an effect on the proportion of populations reached by DFTD at a transmission probability of 0.3, with a negligible influence at higher values (Figure 3).

### 3.2. Spatial Spread of DFTD across Tasmania Is Explained by Metapopulation Dynamics

**Figure 4:**
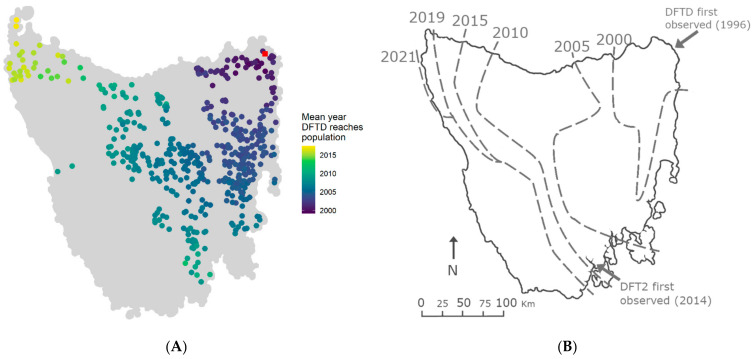
DFTD arrival wave across Tasmania is well approximated by the mechanistic metapopulation model. (**A**) Year of DFTD arrival at each local population based on averaged outputs from metapopulation simulations using the parameter combination that best matches empirical data (δ = 0.1, *γ*_0_ = 0.009 and β = 0.4). The initially infected population at the beginning of simulations is represented by a red square. Point colour represents the year of DFTD arrival; grey points are populations that DFTD did not reach within 25 years of disease introduction. (**B**) Map of the estimated disease front in Tasmania based on empirical data analysis (modified from [47]). The map represents the data used to match simulation results and identify the parameter combination used to obtain results presented in (**A**).

We compared our simulation results to recent empirical DFTD disease front predictions in order to understand the metapopulation mechanisms that might be behind DFTD spread. Out of all parameter combinations explored, the scenario that most closely matched the observed DFTD disease front (Figure 4B) was that of contact distance = 0.1, baseline dispersal rate = 0.009 and transmission probability = 0.4. In this scenario, 67.5% of populations fell within the correct disease arrival wave (i.e., matched the year of arrival reported in previous research [47]). The results for disease arrival wave matching for the five scenarios that most and least matched the observed disease front are presented in Supplementary Materials Table S2. The mean year of arrival of DFTD at each local population from simulations using these parameter values resembled empirical observations, only reaching the north-western corner of Tasmania within the last years of the simulation (Figure 4A).

**Figure 5:**
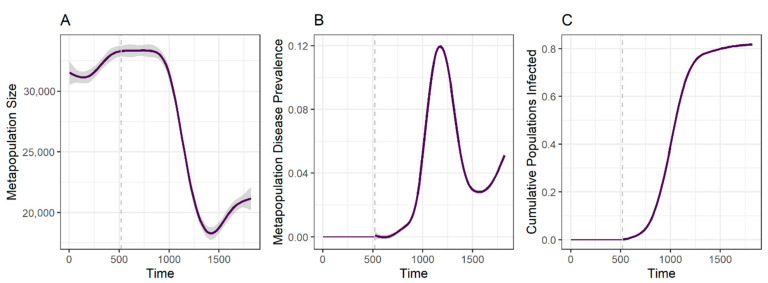
Metapopulation dynamics of disease spread matching the empirically observed disease spread front. Total metapopulation size (**A**), metapopulation-level DFTD prevalence (**B**), and cumulative fraction of populations reached by DFTD (i.e., have had at least one diseased individual during the course of the simulation) (**C**) through time. Vertical grey dashed lines indicate time of DFTD introduction. Lines and shadows around them represent mean and standard error values across 20 replicated simulations with parameters δ = 0.1, *γ*_0_ = 0.009 and β = 0.4).

Metapopulation size and disease prevalence of simulations with this parameter combination exhibit a strong decline and increase, respectively, up until approximately 700 weeks after DFTD introduction (~week 1200), with signs of a metapopulation recovery after that (Figure 5). The final increase in cumulative populations infected after the brief plateau corresponds to the north-western populations, which only become infected towards the end of the simulation, with the disease front progressing more slowly than in previous years. These patterns resemble those observed from empirical data during comparable time periods (Figure 5 in [27]).

### 3.3. Fragmentation Reduces Disease Spread at the Cost of Population Size

**Figure 6:**
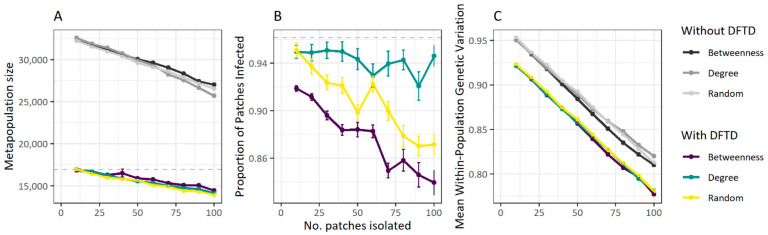
Effects of fragmentation on DFTD spread in Tasmanian devil metapopulations. Metapopulation size (**A**), proportion of individuals infected within the metapopulation (**B**) and mean within-population genetic diversity index (**C**) are influenced by the number of populations isolated from the metapopulation. Proportion of populations infected is also influenced by whether populations are isolated based on the population’s centrality in the metapopulation (i.e., betweenness), its connectivity (i.e., degree) or at random (**B**). Colours represents different population isolation methods. Points and vertical bars display the average and standard error of the different quantities across 20 replicated simulations using the parameter combination that best matches the spatial wave of disease spread (δ = 0.1, *γ*_0_ = 0.009 and β = 0.4); Figure 4.

Metapopulation fragmentation experiments resulted in similar magnitudes of total metapopulation size decline with increasing numbers of populations being isolated regardless of whether populations were isolated at random or according to their position in the metapopulation network (degree or centrality) (Figure 6A). The proportion of populations reached by DFTD varied between population isolation methods. Isolation according to betweenness centrality or at random resulted in declines in the number of populations with DFTD presence as the number of isolated populations increased. Population isolation by degree (i.e., number of connected neighbouring populations) did not show such a relationship (Figure 6B).

As expected, within-population genetic diversity also decreased as the number of populations isolated increased for all population isolation methods (Figure 6C). These patterns were also observed in simulations without DFTD, although both metapopulation size and population genetic diversity were considerably higher in the absence of DFTD, illustrating that population isolation does not curtail the negative impact of DFTD at the metapopulation level (Figure 6).

## 4. Discussion

We developed an individual-based metapopulation model that allowed us to investigate the synergistic effects of local-scale disease transmission processes and population dynamics (e.g., transmission rate and inter-individual contact distance), and landscape-scale metapopulation processes (e.g., dispersal), on the spread of a transmissible cancer in Tasmanian devil populations, with the consequences for population abundances that ensue. We matched the outcomes of this model to published empirical data and identified parameter combinations able to reproduce observed patterns of disease spread. We further used this information to perform *in-silico* metapopulation fragmentation experiments to investigate the potential effects of population isolation measures on DFTD spread and its consequences for population persistence and dynamics. Our results suggest that inter-individual contact distance, a measure of within-population mixing and contact facilitating disease transmission, the dispersal of individuals among local populations and DFTD transmission rates interact in non-intuitive ways to drive population declines in Tasmanian devils.

Whereas dispersal was identified as the main driver of regional DFTD spread, this was only observed for relatively high DFTD transmission rates (≥0.3). The relevance of inter-individual transmission for effective spread is reinforced by our results for intermediate transmission rates (0.3 and to some extent 0.4). These levels of transmission alone are still not high enough to ensure complete spread, unless contact distance between individuals is high enough to increase transmission events. These observations hint at the critical role of within-population mixing and transmission rate in determining DFTD prevalence, and associated individual death, at the local population level. This would eventually translate into an overflow of disease into neighbouring populations, creating a source–sink phenomena, and ultimately resulting in regional disease spread.

The implication that local within-population mixing and dispersal probability both influence the magnitude of a regional outbreak means that a management method that targets either one could prevent a local outbreak from progressing. We hypothesised that isolating some local populations would reduce disease spread, resulting in a larger population size, but at the risk of decreasing population mixing and the potential associated loss of genetic diversity. While we predicted correctly that population mixing would decrease leading to lower within-population genetic diversity, population size also declined as the number of isolated patches increased, and the proportion of populations that DFTD reached varied depending on the population isolation method. Isolating populations with the highest betweenness was the most effective approach for reducing metapopulation DFTD prevalence, which is in line with previous suggestions that immunising nodes in a network with high betweenness is more effective than immunising those with a high degree [50]. Furthermore, isolating populations with a high degree in our simulations was less effective at reducing DFTD prevalence than isolating at random, which was counter to both our expectations and the results of previous immunisation studies [51]. One explanation as to why isolating populations by their number of connections to neighbouring populations (i.e., degree) had little effect on the spread of DFTD across the metapopulation is that the 100 populations with the highest degree were all clustered in one geographic area where DFTD was already present before the populations were isolated, and so their isolation did not lie in the way of the disease front. In other metapopulation structures where this clustering pattern is not seen, we would expect isolation by degree to have a greater influence on the course of the outbreak [51]. The place of introduction of an emerging disease, the landscape context and also the timing of any spatial intervention strategy in relation to previous disease spread may therefore determine which spatial isolation methods may be most efficient.

We found that the number of devils living in populations that were isolated declined rapidly, reducing to zero in some cases (Figure S4). Due to their isolation, these populations were unable to be recolonised by individuals from neighbouring populations, suppressing, thus, the “rescue effect” commonly observed in metapopulation dynamics [52,53] and highlighting its importance for devil population persistence. On the other hand, populations that remained within the metapopulation network were quickly recolonised if their population size dropped to zero (Figure S5). As we increased the number of isolated populations, more of these non-recolonisable population extinctions occurred. It has long been argued whether increased connectivity between populations is beneficial or harmful during a disease outbreak, with some arguing that wildlife corridors could facilitate the spread of disease [54], and other studies showing that local populations benefit from recolonization by individuals from neighbouring populations [55,56], via the “rescue effect” [52,53]. In our simulations, while DFTD indeed spread further in more highly connected metapopulation structures, isolating populations, suppressing, thus, recolonisation, caused more harm to the devil population than the potential benefits of reducing disease spread would confer. This result also supports Siska et al.’s [39] suggestion that population recolonisation is an important factor in preventing devil extinction. Together these findings suggest that trying to completely isolate some populations to reduce the long-distance movement of devils would not be a suitable method to prevent further spread of an ongoing regional DFTD outbreak, and that while Hess [54] was correct in correlating increased metapopulation connectivity with increased disease spread, the benefits of this connectivity far outweighs the risks in this scenario. Population isolation through fencing may be unsuitable for devils as the disease is already present throughout the species distributional range. The only area where the disease has not been detected is southwestern Tasmania’s temperate rainforest and button grass (sub-optimal habitat for the species), where fencing is not logistically feasible. Isolation may be a viable management method when accompanied with efforts to actively maintain the size and genetic diversity of isolated populations, or in species where population size and genetic diversity is less of a concern.

Vaccination has been proven to be effective in reducing disease spread in other wildlife diseases, such as rabies [57]. The release of captive-bred, immunised devils into local populations has been previously suggested to be a viable option in reducing DFTD spread [37]. Devils have been injected with sonicated DFTD cells with the aim to stimulate adaptive immunity as a potential vaccine [58], however, there is no evidence that attempted immunizations are prophylactic in the wild [59,60]. Furthermore, empirical and theoretical evidence suggest that vaccinations that do not prevent transmission and spread of disease (often referred to as leaky or imperfect vaccines) can create ecological and epidemiological conditions that would allow more virulent pathogen strains to emerge and persist [61,62]. In the case of the Tasmanian devils and DFTD, an increasing number of studies have demonstrated natural adaptations to the epidemic [63,64], with devils developing defence mechanisms against infection, such as immune responses against DFTD, the upregulation of a tumour suppressor gene [65,66] and genetic changes in the tumour, leading to reduced transmission and epidemic outcomes [40,67]. Future research should integrate these devil-tumour evolutionary processes to evaluate their long-term effects on disease spread and metapopulation dynamics.

While it is too late to prevent DFTD from spreading across Tasmania, it is hoped that alternative management options can be developed to prevent the spread of a second and independently-evolved transmissible cancer affecting Tasmanian devils, devil facial tumour 2 (DFT2) [68], from causing another wave of catastrophic population decline [69,70]. DFT2 is so far confined to the geographic peninsula where it was discovered in 2014, in south-eastern Tasmania [69]. Although there is currently very little information on the epidemiology and population effects of DFT2, localised spatial spread of DFT2 has been found to be much slower than in DFTD [69]. DFTD has spread throughout Tasmania over continuous habitat at a rate of 25 km per year [34]; however, DFT2 is confined to a peninsula bounded by water on the sides (east, west and south), and a highly urbanised landscape to the north, which has resulted in a spatial spread of 7 km per year [69]. Another factor contributing to the slow spread of DFT2 might be the current low devil population densities across Tasmania, combined with human modified and fragmented landscapes in the surrounding areas where DFT2 emerged. Future models could integrate DFTD–DFT2 infection dynamics and assess the role of highly urbanised–fragmented landscapes on disease spread, and their effects on metapopulation dynamics.

Counter to empirical observations where disease prevalence is observed to remain high behind the disease front [33], our model predicts a peak in DFTD cases that begins to decline shortly after the devil metapopulation size starts to decline, and rises again when the metapopulation begins to recover, likely due to the initial reduction in DFTD prevalence. This pattern was also observed by Wells et al. [41]. One explanation as to this departure from field observations is that both models use density- rather than frequency-dependent DFTD transmission, and that the existence of a host density threshold could cause this reduction in disease prevalence. This explanation is supported in our results by the resurgence of DFTD prevalence that occurs once the metapopulation size starts to recover. One line of evidence suggests strong evidence for frequency-dependent transmission [30], whereas a more recent study also supports the density dependence of spread [27]. It is likely that these two modes of transmission are not exclusive and that density thresholds for the disease to take off might exist. Future versions of our model could, thus, incorporate a combination of frequency- and density-dependent transmission to investigate whether this can reproduce the observed patterns of DFTD persistence. We also observed the metapopulation size decreasing as dispersal probability increased, even in the absence of DFTD. It is likely that the increased dispersal of individuals results in source–sink dynamics in which well-connected populations act as pseudo-sinks (populations that are net receivers of dispersers but do not necessarily depend on them to remain viable or increase their relative contribution as sources) [23], as previously observed by Zamberletti et al. [71], and so are pushed above their carrying capacity, resulting in an increased death rate (Supplementary Materials Figure S6).

Our model also does not consider seasonal changes in transmission, social networks of devils or the presence of DFT2, all of which may be beneficial to include in future models of disease spread and persistence. However, such a model could be used in the future to optimize alternative management strategies and forecast future disease dynamics in the devil-DFTD-DFT2 system. Our work highlights the view that host-pathogen theory, population and ecological processes can be applied to reveal the dynamics of infectious cancers in wildlife and further understand how populations and species respond to oncogenic processes. Furthermore, it emphasizes the need to consider what potential harm could result from spatial intervention strategies, and whether this harm would be an acceptable price to pay for the reduction in disease spread.

## Data Availability

All of the source code developed to implement the model and run the model simulations to produce the results presented in this study has been uploaded to the GitHub repository: https://github.com/RowanDurrant/DFTD (accessed on 12 November 2021).

## Supplementary Materials

The following are available online at https://www.mdpi.com/article/10.3390/pathogens10121592/s1, Figure S1: Distribution of carrying capacities and patch area sizes resulting from the calculations of habitat patch areas and population sizes extracted from vegetation maps and density estimates, Figure S2: Distribution of infection to death time values (in weeks) for individuals tracked over the course of an entire simulation, Figure S3: Metapopulation decline as dispersal probability increases in the absence of DFTD, Figure S4: Example of the population decline observed in local populations after they are isolated from the rest of the metapopulation, Figure S5: Comparison between isolated and non-isolated populations when the population size reduces to zero, Figure S6: Example comparison of population size relative to carrying capacity for the most and least connected populations over time, Table S1: Description and values of the parameters used in the individual-based metapopulation model of Tasmanian devil DFTD spread, Table S2: Fraction and number of populations correctly matching the disease arrival wave and parameter value combinations corresponding to these outcomes, Methods S1: Full description of the methods.
