# Supplementary material for: Disruption of Metapopulation Structure Reduces Tasmanian Devil Facial Tumour Disease Spread at the Expense of Abundance and Genetic Diversity
Source: Pathogens. 2021 Dec 8;10(12):1592. doi: 10.3390/pathogens10121592 (PMC8705368; doi:10.3390/pathogens10121592)
Supplement: Supplementary file 1 [file pathogens-10-01592-s001.zip › pathogens-1483701-supplementary.pdf]

pathogens

MDPI

Supporting Information:

Disruption of Metapopulation Structure Reduces Tasmanian Devil Facial Tumour Disease Spread at the Expense of Abundance and Genetic Diversity

Rowan Durrant, Rodrigo Hamede, Konstans Wells and Miguel Lurgi

Supplementary S1: Full description of the methods

Generation of the Metapopulation structure

Land cover data was obtained from the digital vegetation map of Tasmania, TASVEG version 2 (TASVEG2) [1]. Only habitat patches ≥ 5 km2 in size were considered. This procedure removed any patches where a local devil population was unlikely to persist due to restricted habitat availability, while maintaining a connected metapopulation network. Predicted devil density of each local population was extracted from recently published density estimates [2]. We overlaid the TASVEG2 vegetation map over Cunningham et al.'s density estimates map for the year 1986 (10 years prior the estimated appearance of DFTD in Tasmania, and the starting point of our simulations) and extracted the mean devil density over the area of each habitat patch. These local population estimates were used as the initial population numbers at the start of the model simulations. Any populations with a devil density below a threshold of 0.5 devils/km^2 were excluded from the metapopulation. This yielded a total of 477 populations, with a mean habitat patch area of 13.3 km^2 (minimum area 5.0 km^2, maximum area 102.2 km^2).

Individual-based model: Demographic and epidemiological processes

Reproduction: Once a year (i.e. 1 every 52 weeks), female individuals of breeding age (i.e. ≥ 52 weeks old) were randomly selected with a probability of reproducing per breeding season that was determined based on their age (see *Reproduction Probability* in Supplementary S1:Table S1 for specific probabilities across age groups). Females were not able to reproduce if there were no adult males present within the same local population. Number of offspring recruited as free-roaming individuals from reproducing females was drawn from a binomial distribution (number of events = 4, probability of success = 0.72) according to empirical observations of pouch young survival rates [3]. Each offspring released into the free-roaming population was assigned an age between 32 and 36 weeks with equal probability to account for the duration of the yearly breeding season. This way of simulating the breeding season allowed for computational efficiency (i.e. only one reproductive event per year) while yielding realistic estimates of breeding season duration (i.e. heterogeneity in the aging of offspring released into the population). Furthermore, this approach does not impact disease dynamics as the behaviour of pouch and den young is assumed to be irrelevant to the spread of DFTD. Each offspring was assigned coordinates within their local population equal to those of their mother, sex assigned randomly with equal probability, and a genotype only for population isolation simulations (see "*Tracing the mixing of individuals from different populations*").

Local movement: Movement direction and distance of individual devils within their local population were randomly determined each time step. Direction was randomly sampled from a normal distribution between 0 and 2*π, whereas distance was normally distributed around the average movement speed (mean = 5 kilometres per week, s.d. 0.5). If their movement distance in one direction exceeded the boundaries of the spatial area of the local population, the devil would stop at this boundary. DFTD transmission between individuals was influenced by the maximum pairwise Euclidean distance over which

devils were assumed to interact within the weekly time widow (hereafter referred to as the inter-individual contact distance, δ). Larger contact distances would translate into higher levels of within-population mixing, a measure relevant for disease spread.

Dispersal: Individuals disperse to neighbouring populations (i.e. connected via a link in the metapopulation) with a certain baseline density-dependent dispersal probability (γ_0; Supplementary S1:Table S1) and proportional to population size with its maximum at carrying capacity thus:

$$\gamma_p = \gamma_0 * \min\left(\frac{N_{p,t}}{K_p}, 1\right) \qquad (S1)$$

where γ_p is the dispersal probability for devils within population *P*, γ_0 is the baseline dispersal probability, $N_{p,t}$ is the population size within population *p* at time *t*, and K_p is the carrying capacity of population *p*. Due to increased rates of dispersal being observed in juvenile devils (under 52 weeks old) [4], these individuals were assumed to have a dispersal probability 10 times higher than adults. Dispersal values used are estimates based on the limited empirical evidence available [4] and initial testing in simulations to produce a range of rates of regional disease spread that encompass the observed rate of DFTD spread across Tasmania.

Aging: Each time step of the model simulation, one week was added to every individual's age.

Non-DFTD deaths: Population growth was limited by the local populations' carrying capacity (*K*). Carrying capacity of each local population was calculated using devil density estimates from [2]. For each year between 1985 and 1996 (the emergence of DFTD), the TASVEG2 vegetation map was overlaid over Cunningham *et al.*'s density estimates maps and the mean devil density over the area of each habitat patch was extracted. The largest density estimate per patch across these years was used to calculate the corresponding population's carrying capacity. Local carrying capacities were uniformly scaled to reach a metapopulation-level carrying capacity of 53,000 individuals (the pre-DFTD maximum population size estimates [2] (Supplementary S1:Fig. S1).

Individual death rate (μ) was set to a constant when populations were below *K* (μ_c, Supplementary S1:Table S1), increasing with local abundance as the number of individuals in the population exceeded *K*, up to a maximum death rate (μ_{max}) of 0.012 (the maximum death rate value that results in a stable metapopulation size in the absence of DFTD):

$$\mu = \min\left(\mu_c + 0.005 * \left(N_{p,t} - K_p\right), \mu_{max}\right) \text{ if } N_{p,t} \geq K_p; \quad \mu_c \text{ otherwise} \qquad (S2)$$

where μ_c is the default death rate for the individual's age category (see Supplementary S1: Table S1 for values). Additionally, individuals that reached the maximum age of 364 weeks were considered dead and removed from the system.

Infection: The force of infection (λ_i) is the probability of an individual becoming newly infected with DFTD in a given week, and is defined as:

$$\lambda_i = min\{(\sum d_{ij} * \frac{V_j}{V_{max}} * \beta_i * \beta_j), 1\} \qquad (S3)$$

where d_{ij} is a Boolean indicator of whether a potentially infective individual *j* is within the contact distance (δ) of the susceptible individual *i*, V_j is the tumour volume of individual *j*, V_{max} is the maximum possible tumour volume, and the disease transmission coefficients β_i and β_j represent the probability of antagonistic interaction leading to disease transmission based on individual *i* and *j*'s age. If the susceptible individual becomes infected during an interaction, it was seeded with a tumour of volume 0.0001 cm^3 (the minimum tumour volume at the onset of growth).

Tumour growth: Increase in tumour volume for each individual per week was determined using a logistic function, adapted from Hamede *et al.* (2017):

$$V_t = \frac{V_{max}}{\left[1 + \left(\frac{V_{max}}{V_{t-1}} - 1\right) * \exp(-r)\right]} \quad (S4)$$

where V_t is tumour volume in the current weekly time step, V_{t-1} is the tumour volume in the previous time step, V_{max} is the maximum tumour volume, and r is tumour growth rate in cm^3 per week (Supplementary S1: Table S1).

DFTD-induced mortality: Even though data on devils' causes of mortality is sparse, previous studies have found that DFTD-induced mortality is close to 100%, with animals succumbing to the disease within 6 to 18 months [6,7]. For simplicity, we assume a 100% mortality rate. Using individual-based simulated mortality data from our model we estimated the tumour volume-dependant mortality rate to ensure a mean infection-to-death time period of 35 weeks (well within the estimates from the empirical data) (Supplementary S1:Fig. S2). Thus, disease-induced mortality Ω_{size} per time step was assumed to be proportional to the individual's tumour volume and given by:

$$\Omega_{size} = \left(\frac{V_t}{V_{max}}\right)^4 \quad (S5)$$

If an individual was selected to die in that time step, it was removed from the population.

Table S1: Description and values of the parameters used in the individual-based metapopulation model of Tasmanian devil DFTD spread.

Parameter	Symbol	Description	Values	Reference
Initial population size		Number of devils within each population at the start of each simulation.	19 - 636 individuals according to patch size (median: 48; mean: 75.65; s.d.: 81.7; total metapopulation: 36,086)	Extracted from [2]'s 1986 density estimates as an average across densities within each habitat patch (see *Methods*).
Carrying capacity	K	Maximum size of devils' local populations. Death rate increases above this number.	28 - 914 individuals according to population size (median: 71; mean: 111.1; s.d.: 119.1; total metapopulation: 52,996)	Calculated based on the max density estimates between 1985 and 1996 from [2] for each habitat patch (see *Methods*).
Breeding age		Minimum and maximum age when devils can reproduce.	Minimum 52 weeks, maximum 260 weeks	[8]
Maximum age		Maximum age of devils before being removed from the system.	364 weeks	[7]
Offspring age		Age of offspring recruited as free-roaming individuals into the population.	32 – 36 weeks	[3]
Reproduction rate (females)		Probability of a mature female reproducing during the annual breeding season depending on age (age categories: A1: under 52 weeks, A2: 52 – 104 weeks, A3:104 – 156 weeks, A4: 156 - 260 weeks, A5: over 260 weeks).	A1 = 0; A2 = 0.21; A3 = 0.71; A4 = 0.56; A5 = 0	[7] (approximate parameter estimates).
Maximum litter size		Maximum number of offspring each female can produce in a breeding season.	4	[3]
Pouch young recruitment rate		Recruitment rate of pouch young as free-roaming offspring.	0.72	
Baseline dispersal probability	γ_0	Probability of dispersing to a neighbouring population per weekly time step.	Values between 0.001 – 0.01, increasing in increments of 0.001	Range of selected values that resulted in spatial disease spread matching empirical evidence.

Movement distance		Distance travelled by a single individual within its local population per weekly time step.	5 km	Within range of parameters used in [7].
Minimum tumour volume		Minimum tumour volume at onset of growth.	0.0001 cm^3	[9]
Maximum tumour volume	V_{max}	Asymptotic tumour volume as used in logistic growth curve.	202 cm^3	
Tumour growth rate	r	Scale parameter of logistic growth curve of tumours, given as value of weekly growth.	0.448	
Contact distance	δ	Distances within which two devils can potentially interact and transmit DFTD.	0.1 – 0.5 km, (increasing in increments of 0.1 km)	Adjusted from values within range used in [7] to account for increased devil density.
Disease transmission coefficients	β	Assumed probability of engaging in an antagonistic interaction that leads to disease transmission between devils within contact distance (δ).	0.2 – 0.8 (increasing in increments of 0.1); 0 for devils < 52 weeks of age	[7] (approximate parameter estimates). No juvenile devils have been observed with DFTD [10].
Default death rate	μ_c	Assumed death rate per weekly time step (age categories as above).	A1 = 0.011; A2 = 0.004; A3 = 0.006; A4 = 0.005; A5 = 0.012	Parameter values estimated in [7]
Maximum death rate	μ_{max}		0.012	Arbitrary value to ensure population size stability in the absence of DFTD.

Table S2. Fraction and number of populations correctly matching the disease arrival wave and parameter value combinations corresponding to these outcomes. The top and bottom 5 parameter combinations ranked according to the degree of matching to the disease arrival wave are shown. Combinations constrained to those where the final metapopulation size is within the confidence intervals of Cunningham et al.'s [2] 2020 predictions (12,500 – 23,100 individuals).

Contact distance	Dispersal probability	Transmission probability	No. populations matching disease arrival wave	Percent populations matching disease arrival wave
0.1	0.009	0.4	322	67.51
0.1	0.008	0.4	319	66.88
0.3	0.006	0.4	310	64.99
0.2	0.008	0.4	309	64.78
0.1	0.01	0.4	306	64.15
0.3	0.002	0.4	6	1.26
0.3	0.001	0.7	7	1.47
0.4	0.001	0.5	7	1.47
0.4	0.009	0.3	10	2.10
0.1	0.003	0.4	11	2.31

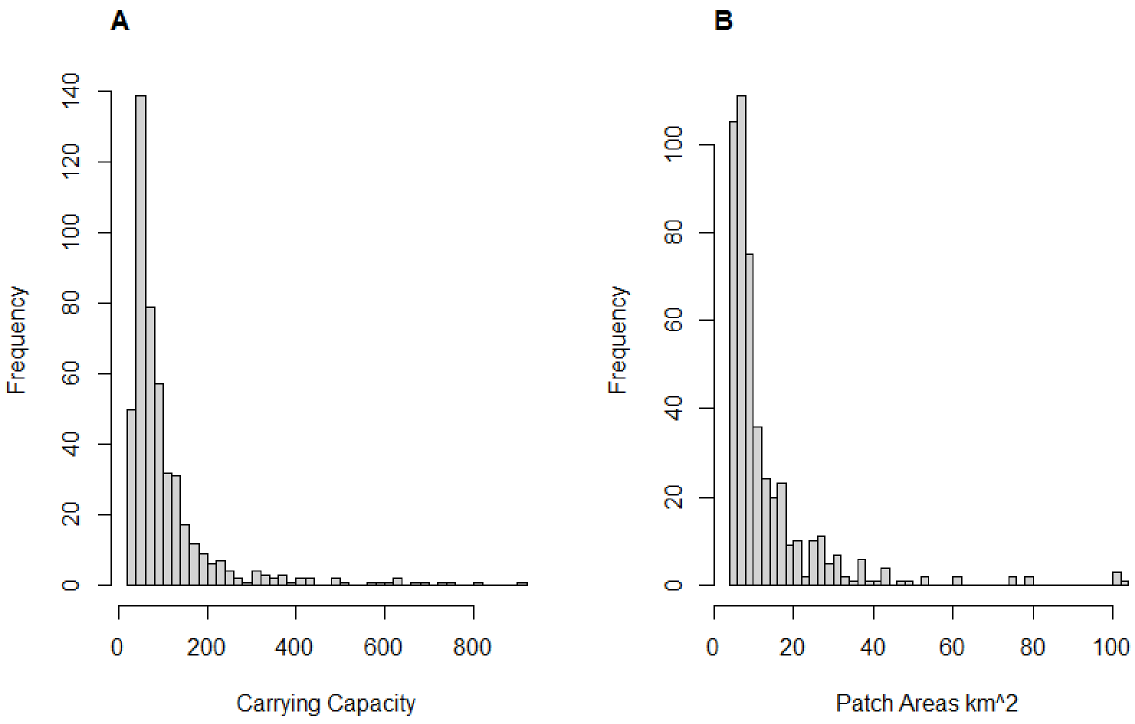

Figure S1. Distribution of carrying capacities and patch area sizes resulting from the calculations of habitat patch areas and population sizes extracted from vegetation maps and density estimates (see *Supplementary S1: Full description of methods*).

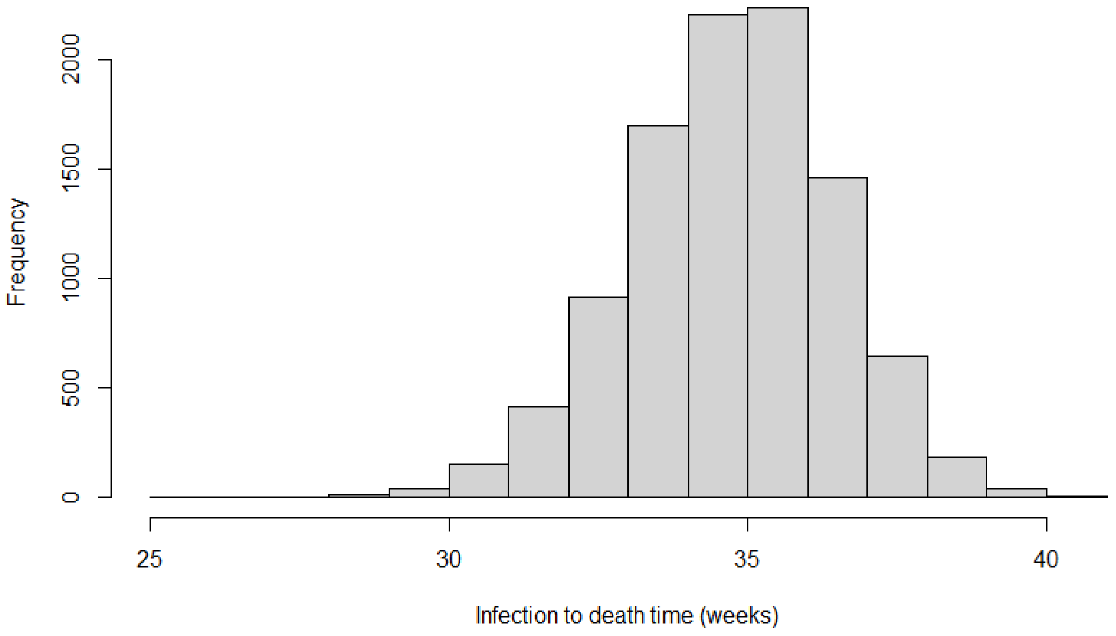

Figure S2. Distribution of infection to death time values (in weeks) for individuals tracked over the course of an entire simulation using equation (Eq. S5) to calculate death rate (see *Supplementary S1: Full description of methods*).

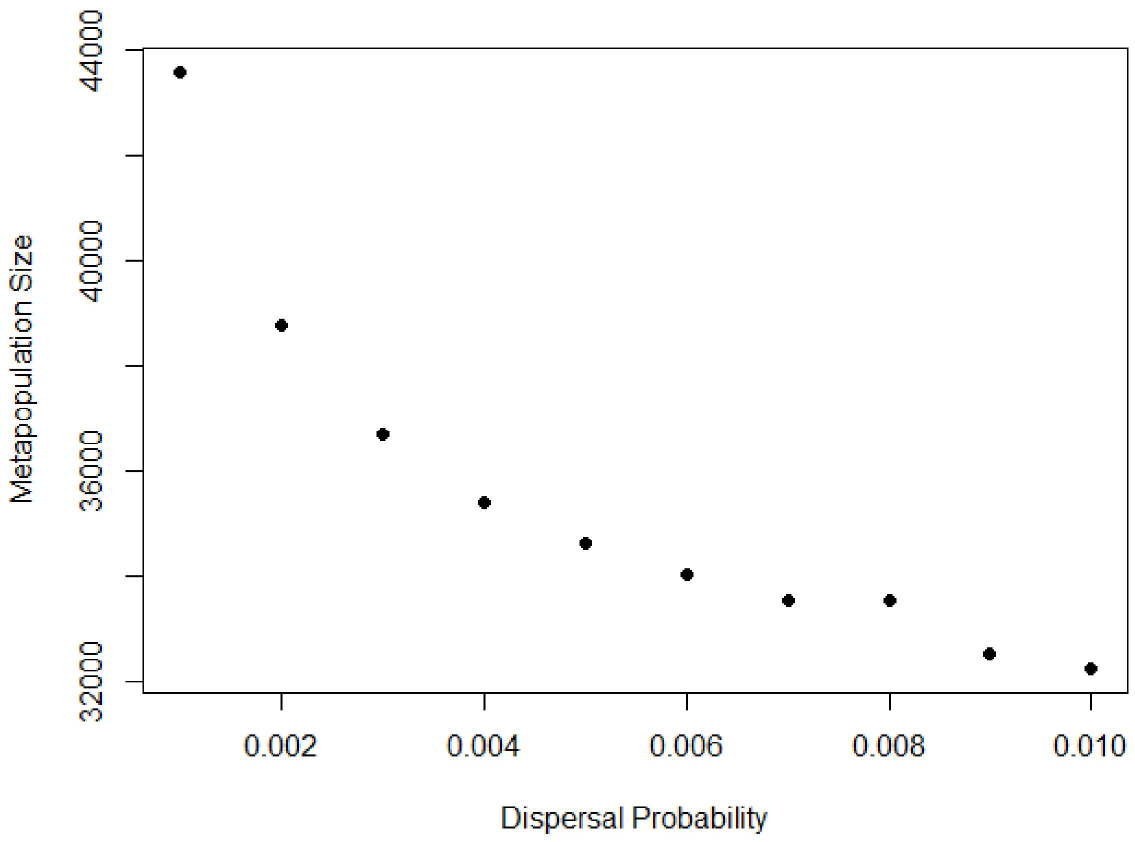

Figure S3. Metapopulation decline as dispersal probability increases in the absence of DFTD.

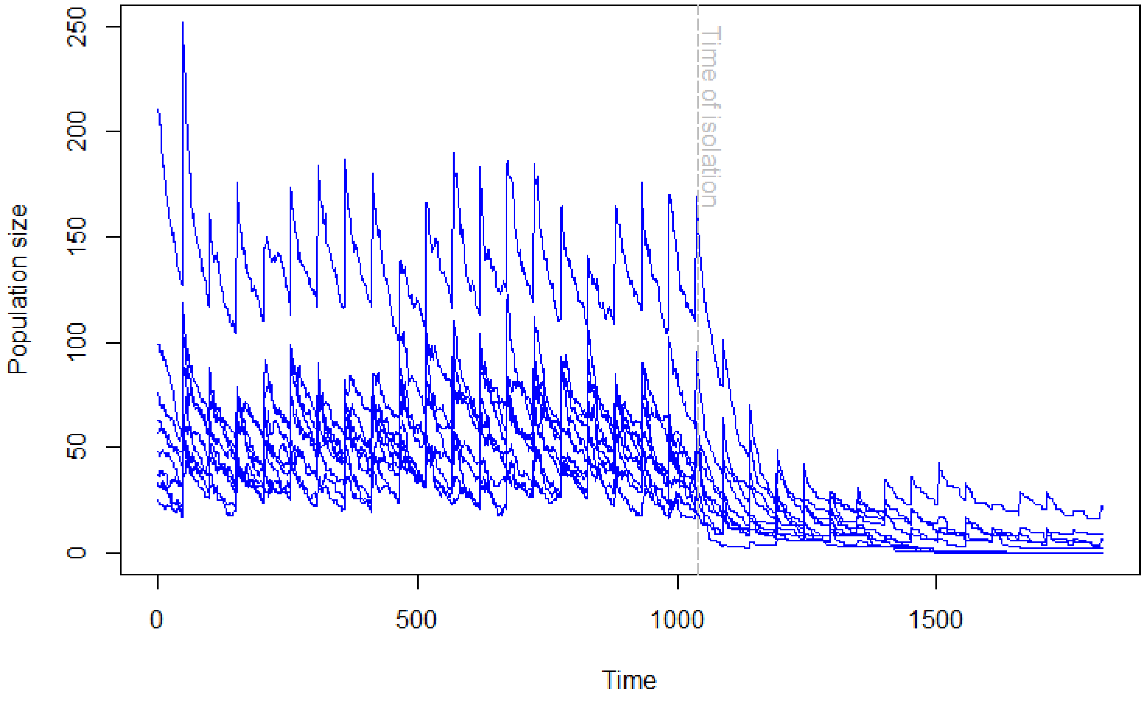

Figure S4. Example of the population decline observed in local populations after they are isolated from the rest of the metapopulation. Data in plot corresponds to a randomly selected isolation simulation replicate. All isolated populations (n = 10) are plotted.

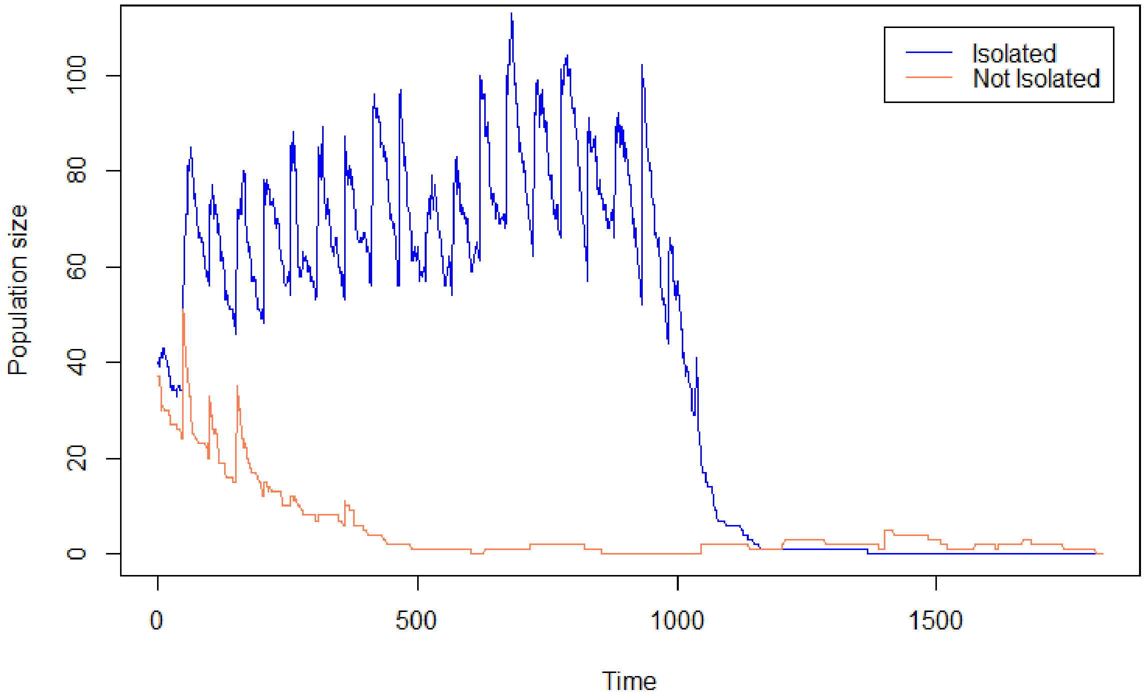

Figure S5. Comparison between isolated and non-isolated populations when the population size reduces to zero. The non-isolated population can become recolonised (but continues in an extinction-reintroduction cycle), whereas the isolated population cannot become repopulated. Data plotted corresponds to a randomly selected isolation simulation replicate. All populations where the population size reaches zero are plotted. In this instance only one local population of each kind (isolated and non-isolated) reached zero, but the pattern of the population trajectory is general across all simulation replicates.

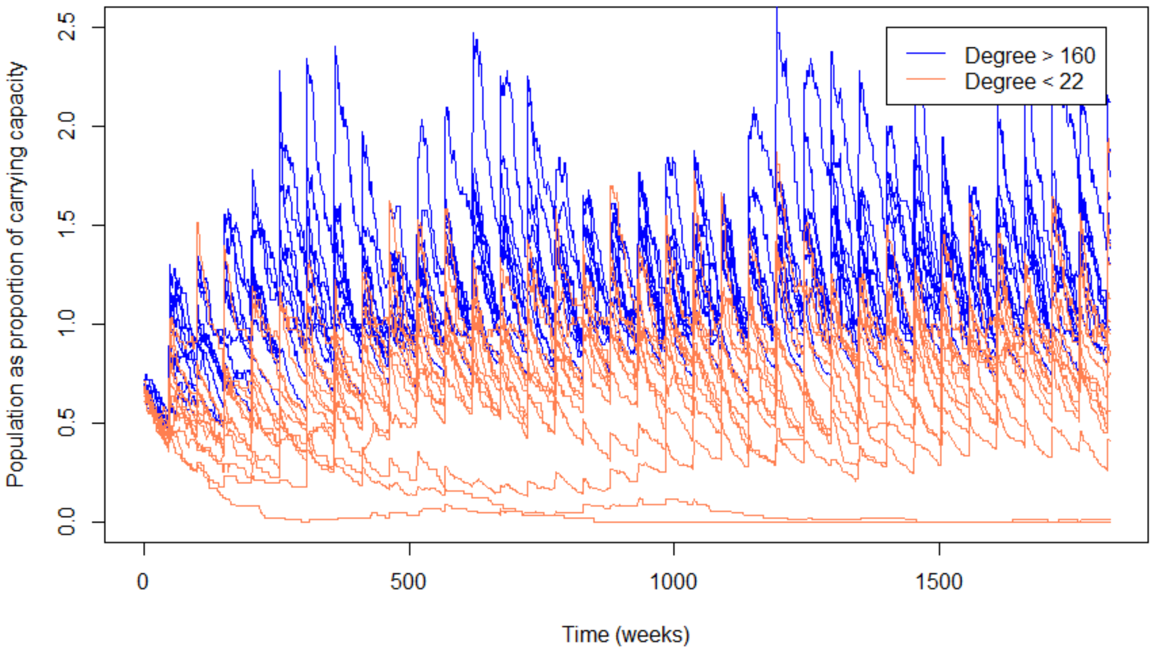

Figure S6. Example comparison of population size relative to carrying capacity for the most and least connected populations over time. More highly connected populations are consistently above their carrying capacity, suggesting that they are acting as pseudo-sinks, as discussed in [11].

References

[1] Department of Primary Industries, Parks, Water and Environment (Tasmania). TASVEG 2.0 2009.
[2] Cunningham CX, Comte S, McCallum H, Hamilton DG, Hamede R, Storfer A, et al. Quantifying 25 years of disease-caused declines in Tasmanian devil populations: host density drives spatial pathogen spread. Ecol Lett 2021;24. https://doi.org/10.1111/ele.13703.
[3] Guiler ER. Observations on the Tasmanian Devil, Sarcophilus harrisii (Marsupialia : Dasyuridae) II. Reproduction, breeding and growth of pouch young. Aust J Zool 1970;18:63–70. https://doi.org/10.1071/zo9700063.
[4] Lachish S, Miller KJ, Storfer A, Goldizen AW, Jones ME. Evidence that disease-induced population decline changes genetic structure and alters dispersal patterns in the Tasmanian devil. Heredity 2011;106:172–82. https://doi.org/10.1038/hdy.2010.17.
[5] Hamede RK, Beeton NJ, Carver S, Jones ME. Untangling the model muddle: Empirical tumour growth in Tasmanian devil facial tumour disease. Sci Rep 2017;7. https://doi.org/10.1038/s41598-017-06166-3.
[6] Hamede R, Lachish S, Belov K, Woods G, Kreiss A, Pearse A-M, et al. Reduced Effect of Tasmanian Devil Facial Tumor Disease at the Disease Front. Conserv Biol 2012;26:124–34. https://doi.org/10.1111/j.1523-1739.2011.01747.x.
[7] Wells K, Hamede RK, Jones ME, Hohenlohe PA, Storfer A, McCallum HI. Individual and temporal variation in pathogen load predicts long-term impacts of an emerging infectious disease. Ecology 2019;100:e02613. https://doi.org/10.1002/ecy.2613.
[8] Jones ME, Cockburn A, Hamede R, Hawkins C, Hesterman H, Lachish S, et al. Life-history change in disease-ravaged Tasmanian devil populations. Proc Natl Acad Sci 2008;105:10023–7. https://doi.org/10.1073/pnas.0711236105.
[9] Wells K, Hamede RK, Kerlin DH, Storfer A, Hohenlohe PA, Jones ME, et al. Infection of the fittest: devil facial tumour disease has greatest effect on individuals with highest reproductive output. Ecol Lett 2017;20:770–8. https://doi.org/10.1111/ele.12776.
[10] Hawkins CE, Baars C, Hesterman H, Hocking GJ, Jones ME, Lazenby B, et al. Emerging disease and population decline of an island endemic, the Tasmanian devil Sarcophilus harrisii. Biol Conserv 2006;131:307–24. https://doi.org/10.1016/j.biocon.2006.04.010.
[11] Zamberletti P, Zaffaroni M, Accatino F, Creed IF, De Michele C. Connectivity among wetlands matters for vulnerable amphibian populations in wetlandscapes. Ecol Model 2018;384:119–27. https://doi.org/10.1016/j.ecolmodel.2018.05.008.
